# Are tumor size changes predictive of survival for checkpoint blockade based immunotherapy in metastatic melanoma?

**DOI:** 10.1186/s40425-019-0513-4

**Published:** 2019-02-08

**Authors:** Meihua Wang, Cong Chen, Thomas Jemielita, James Anderson, Xiaoyun (Nicole) Li, Chen Hu, S. Peter Kang, Nageatte Ibrahim, Scot Ebbinghaus

**Affiliations:** 1 Merck & Co., Inc., Kenilworth, NJ USA; 2 Sidney Kimmel Comprehensive Cancer Center at Johns Hopkins, Baltimore, MD USA; 3 BARDS Early Development Statistics – Early Oncology, 351 North Sumneytown Pike, North Wales, 19454 USA

**Keywords:** RECIST, Early efficacy metrics, Early tumor size changes, Cut-off values, Immunotherapy trials

## Abstract

**Background:**

In oncology clinical development, objective response rate, disease control rate and early tumor size changes are commonly used as efficacy metrics for early decision-making. However, for immunotherapy trials, it is unclear whether these early efficacy metrics are still predictive of long-term clinical benefit such as overall survival. The goal of this paper is to identify appropriate early efficacy metrics predictive of overall survival for immunotherapy trials.

**Methods:**

Based on several checkpoint blockade based immunotherapy studies in metastatic melanoma, we evaluated the predictive value of early tumor size changes and RECIST-based efficacy metrics at various time points on overall survival. The cut-off values for tumor size changes to predict survival were explored via tree based recursive partitioning and validated by external data. Sensitivity analyses were performed for the cut-offs.

**Results:**

The continuous tumor size change metric and RECIST-based trichotomized response metric at different landmark time points were found to be statistically significantly associated with overall survival. The predictive values were higher at Week 12 and 18 than those at Week 24. The percentage of tumor size changes appeared to have comparable or lower predictive values than the RECIST-based trichotomized metric, and a cut-off of approximately 10% tumor reduction appeared to be reasonable for predicting survival.

**Conclusions:**

An approximate 10% tumor reduction may be a reasonable cut-off for early decision-making while the RECIST-based efficacy metric remains the primary tool. Early landmark analysis is especially useful for decision making when accrual is fast. Composite response rate (utilizing different weights for PR/CR and SD) may be worth further investigation.

**Trial registration:**

Clinical trials gov, NCT01295827, Registered February 15, 2011; NCT01704287, Registered October 11, 2012; NCT01866319, Registered May 31, 2013.

**Electronic supplementary material:**

The online version of this article (10.1186/s40425-019-0513-4) contains supplementary material, which is available to authorized users.

## Background

Intuitively, the pattern of tumor size changes is indicative of the therapeutic effects of novel anti-cancer regimens. Currently, tumor response and disease progression are based on Response Evaluation Criteria in Solid Tumors (RECIST) criteria, which were initially developed in the mid of 1990s (RECIST 1.0) to standardize and simplifies response criteria and then modified in 2009 (RECIST 1.1) in the context of cytotoxic treatments [1]. Regulatory authorities accept RECIST as an appropriate guideline for these assessments. Tumor response assessments, e.g., tumor shrinkage (response) or growth (progressive disease), are categorized based on pre-specified thresholds and essentially only capture a snapshot from the entire tumor growth kinetics. For example, a partial response (PR) in target lesions requires at least a 30% decrease from baseline in the sum of diameters of target lesions while progressive disease (PD) requires at least a 20% increase in the sum of diameters of target lesions from the smallest sum on study (nadir). However, such use of tumor size measurements is far from efficient and oversimplifies the complex trajectory of tumor size kinetics.

The concerns over the appropriateness of RECIST in measuring treatment benefit and predicting long-term outcomes in the era of immunotherapy have led to some recent efforts in developing alternative efficacy endpoints based on continuous tumor size data collected over time. For example, continuous tumor measurement–based metrics have been explored in terms of the absolute or relative changes in tumor size [2–7], the averaged overall assessments [8], and time to tumor growth using a mechanistic tumor growth model [9]. These efforts mainly focus on evaluating if short-term (6, 8 or 12 weeks) tumor size kinetics are sufficiently predictive of Overall Survival (OS) such that they may be considered for alternative early-stage efficacy endpoints. For traditional chemotherapy and target therapy trials, it has been demonstrated tumor size changes are predictive of OS [4–12], but do not provide any predictive improvement over traditional RECIST response evaluation criteria (e.g. trichotomized response status) [6–8].

Despite the robust progress in the development of immunotherapies, additional intermediate endpoints may be needed to detect signals of early anti-tumor activity, prioritize compound development or combinations, and interpret early phase study results. With an increasingly competitive and dynamic immune-oncology space, advancing successful therapies and terminating unsuccessful therapies earlier is crucial. In early oncology drug development, RECIST-based Objective Response Rate (ORR) and Disease Control Rate (DCR), and Early Tumor Size Changes (ETSC) are commonly used as early efficacy metrics for early decision-making. The predictive value of these metrics has been demonstrated for traditional chemotherapy and target therapy trials [4–12]. However, for immunotherapy trials, it is unclear whether all these early efficacy metrics are still predictive of long-term clinical benefit [13].

In the era of immunotherapy, it is recognized that the tumor size kinetics are not well captured using the same RECIST categories of tumor responses and disease progression. For example, immune checkpoint inhibitors have exhibited unusual response and progression kinetics, such as durable response, durable stable disease, “pseudo”-progression and/or delayed response. All these patterns are associated with favorable survival [14, 15]. Additionally, the immunotherapy treatment effects on Progression-Free Survival (PFS) are not reliable predictor of OS and survival benefit could be observed in the absence of PFS effect [16]. The unique patterns of response, progression, and survival with immune checkpoint inhibitors in melanoma, lung cancer and other cancers lead to renewed interest in exploring novel intermediate endpoints to obtain an early signal of efficacy and assist in go/no-go decision making [16, 17]. Intuitively, given the atypical pattern of response in patients treated with immunotherapies, continuous tumor sizes measurement instead of RECIST categories may be more sensitive to the underlying dynamic patterns of tumor responses, and thus may be more likely to inform down-stream clinically meaningful events such as OS. While ETSC have been explored for chemo and target therapies [18–22], very few analysis results on ETSC are available based on large immunotherapy datasets.

In this paper, we investigated if ETSC as a continuous measurement and RECIST-based response status were predictive of long-term survival benefit in checkpoint blockade based immunotherapy studies in metastatic melanoma, evaluated if tumor size changes provided any predictive improvement over RECIST based efficacy metrics, and explored the optimal cut-off of ETSC with external validation. The goal is to identify appropriate early efficacy metrics that can be predictive of survival for immunotherapy trials.

## Methods

### Data

In this analysis, we included 3 immunotherapy studies (Keytruda KEYNOTE-001, − 002, − 006) evaluating pembrolizumab in advanced melanoma tumors submitted to FDA as initial or supplemental Biologics License Applications for pembrolizumab in melanoma indication.

KEYNOTE-002 is a randomized, controlled, phase 2 clinical trial comparing two pembrolizumab doses with investigator-choice chemotherapy in patients with ipilimumab-refractory melanoma. Five hundred forty ipilimumab-treated patients were randomized in a 1:1:1 ratio to pembrolizumab 2 mg/kg (*n* = 180) or 10 mg/kg (*n* = 181) every 3 weeks or investigator-choice chemotherapy (*n* = 179). KEYNOTE-006 is a randomized, controlled, phase 3 study comparing two pembrolizumab doses with ipilimumab in patients with advanced melanoma. Eight hundred thirty four ipilimumab naïve patients with advanced melanoma were randomized in a 1:1:1 ratio to receive pembrolizumab (at a dose of 10 mg/kg) every 2 weeks (*n* = 279) or every 3 weeks (*n* = 277) or four doses of ipilimumab (at 3 mg/kg) every 3 weeks (*n* = 278). For KEYNOTE-002 and KEYNOTE-006, the primary endpoints were PFS (defined as the time from randomization to documented disease progression according to RECIST v1.1 by independent central review or death from any cause) and OS (defined as the time from randomization to death from any cause). Tumor assessments were done before starting study treatment (baseline), at week 12, every 6 weeks through to week 48, and every 12 weeks thereafter.

KEYNOTE-001 was a multicenter, open-label, phase Ib study of pembrolizumab for patients with advanced solid tumors, which included multiple melanoma expansion cohorts. There are 655 melanoma patients enrolled (135 from a nonrandomized cohort [*n* = 87 ipilimumab naive; *n* = 48 ipilimumab treated] and 520 from randomized cohorts [*n* = 226 ipilimumab naive; *n* = 294 ipilimumab treated]). Patients received pembrolizumab 10mg/kg every 2weeks, 10mg/kg every 3weeks, or 2mg/kg every 3weeks. Tumor response was assessed every 12 weeks by independent central review using RECIST v1.1.

Detailed eligibility criteria, initial or final study results for the above 3 studies were published previously [23–26]. These articles published in scientific journals are official outcome of these 3 melanoma studies. For this exploratory research, a later data cut-off date was used. All melanoma patients enrolled to the above 3 studies were included for the data analyses except those 179 patients randomized to the investigator-choice chemotherapy arm from KEYNOTE-002. Patients randomized to pembrolizumab 10mg/kg or 2mg/kg were pooled across doses due to lack of difference between these 2 doses. Tumor size measurement and/or RECIST evaluation may not be available at some time points due to disease progression, treatment discontinuation, death or other reasons; therefore, the number of patients to be included in each specific analysis is different. It is worth noting the target lesion measurement in these 3 studies differed from conventional RECIST 1.1 by allowing up to 10 target lesions and up to 5 per organ (RECIST 1.1 only requires up to 5 target lesions and up to 2 per organ).

### Metrics

Two types of efficacy metrics were evaluated: ETSC and RECIST-based response metrics. Tumor size changes are the percentage changes from baseline in the sum of diameters of target lesions. RECIST-based trichotomized metric consists of three categories based on RECIST criteria: Partial Response/Complete Response (PR/CR) vs. Stable Disease (SD) vs. PD. In addition to the best tumor size changes and best response during the study, 3 different time points (Week 12, 18 and 24) were also evaluated for each metric. Patients without tumor assessment information at the landmark time points (with +/− 2 weeks window allowed) were excluded from time-point specific analysis.

### Statistical methods

The Cox Proportional Hazard (PH) model was fit to assess the effect of tumor size changes and RECIST-based response status on OS, separately, adjusting for baseline tumor sizes which is an independent prognostic factor for overall survival in melanoma [27]. Percentages of tumor size changes were fit as a continuous metric assuming a linear relationship between the log Hazard Ratio (HR) and percentages of tumor size changes. RECIST-based response status was fit as a categorical metric with 3 categories PR/CR vs. SD vs. PD. ETSC and RECIST-based trichotomized metrics at three different landmarks (12, 18 and 24 weeks) were explored to determine how early the efficacy metrics could be meaningful. To avoid length-time bias [28], OS was re-calculated from the landmark time point instead of typical OS from post-randomization.

Based on the training datasets including all KEYNOTE-002 and -006 melanoma patients randomized to pembrolizumab or ipilimumab (while excluding patients randomized to the investigator-choice chemotherapy arm from KEYNOTE-002), the cut-off values for tumor size changes to predict survival outcome were explored via tree based recursive partitioning [29]. Recursive partitioning is a nonparametric technique for prediction and classification. Bagged or bootstrapped [30] cut-off values were obtained by averaging estimated cut-off values across 2000 bootstrap samples. Bootstrap provides a way to account for variations and leads to more accurate estimate than using observed data alone. Survival difference based on the chosen cutoff was tested using the validation datasets including KEYNOTE-001 melanoma patients treated with pembrolizumab. Log-rank test, Kaplan-Meier method and Cox PH model were used to assess the impact of the chosen cutoff on OS.

To derive the optimal cut-offs at the select time point, sensitivity analyses were also conducted to assess a range of cut-offs from 30% tumor reduction (− 30%) to 20% tumor increase (+ 20%) using validation datasets. The performance of different cutoffs was further evaluated via sensitivity, Positive Predictive Value (PPV), as well as specificity and Negative Predictive Value (NPV) [31, 32]. Here PPV is the probability of surviving additional time if ETSC is greater than certain cut-offs and it is an indication of sufficient anti-tumor activity that should translate into an overall survival benefit.

### Evaluation criteria

The associations between each metric and survival were assessed through estimated HRs. Akaike information criteria (AIC) was used to measure and compare relative model fits [33]. AIC deals with the trade-off between the goodness of fit of the model and the simplicity of the model. As a measure of relative quality of statistical models for a given set of data, the smaller the AIC value the better the model-fitting.

To determine how well a model/metric discriminates among patients with different outcomes, the c-index [34–36] was used to compare the relative predictive performance of each model/metric. The c-index in the context of survival considers all pairs of individuals. A pair is considered evaluable if one can determine which individual in the pair dies first. The index is the fraction of all evaluable pairs that are concordant. If the “predicted” survival time (probability) is larger for the patient who (actually) lived longer, the predictions for that pair are said to be concordant with the actual outcomes. The c-index ranges from 0.5 to 1.0 where 0.5 indicates no association and 1 indicates perfect association.

## Results

### Variation of tumor size changes

Table 1 and Table 2 summarize ETSC at Week 12 and best tumor size changes during the study. Best tumor size changes were greater than Week 12 tumor size changes. Tumor size changes in early line patients were greater than those in later line patients. The variation (standard deviations column in Table 1 and Table 2) of tumor size changes in early lines appeared to be smaller than those in later lines.

**Table 1:** Tumor size changes in melanoma patients (Ipilimumab-Naive melanoma (KEYNOTE-006))

	Number of Line of Therapy							
	1L				2L			
	*n*	Median	Mean	Standard Deviation	*n*	Median	Mean	Standard Deviation
Pembrolizumab arm								
% of Early Tumor Sizes Changes (~Week 12)	282	−24.2	−19.7	42.5	152	−9.1	−9.0	47.2
Best % of Tumor Size Changes	282	−53.0	−40.8	52.4	152	−36.3	−29.1	57.8
Ipilimumab arm								
% of Early Tumor Size Changes (~ Week 12)	124	7.8	6.6	46.0	61	−1.6	5.3	62.1
Best % of Tumor Size Changes	124	−6.5	−7.0	56.2	61	−9.7	−6.8	70.2

**Table 2:** Tumor size changes in melanoma patients (Ipilimumab-refractory melanoma (KEYNOTE -002))

	Number of Prior Line of Therapy Received							
	1				2 +			
	(2L)				(3L+)			
	*n*	Median	Mean	Standard Deviation	*n*	Median	Mean	Standard Deviation
Pembrolizumab arm								
% of Early Tumor Size Changes (~Week 12)	78	−7.4	−10.0	31.3	200	−0.1	−2.4	43.1
Best % of Tumor Size Changes	78	−11.2	−23.5	43.2	200	−9.4	−16.0	53.5

### RECIST-based response metric

Best response rates were greater than early response rates and response rates in early line patients were greater than those in later line patients (Additional file 1: Table S1 and Additional file 2: Table S2 in the Supplement). For example, for 1L patients receiving pembrolizumab from KEYNOTE-006, the early response rate (PR/CR) at Week 12 was 36% while the best response rate was 45% (third column in Additional file 1: Table S1). Along the same line of patients, the early SD rate at Week 12 was 26% while the best SD rate was 19% (third column in Additional file 1: Table S1); among those patients with early SD, 30% converted to PR later on. The PD rate at early time point (Week 12) appeared similar to that based on best trichotomized response status during the whole study period.

### Comparison of different metrics at different time points

**Fig. 1:**
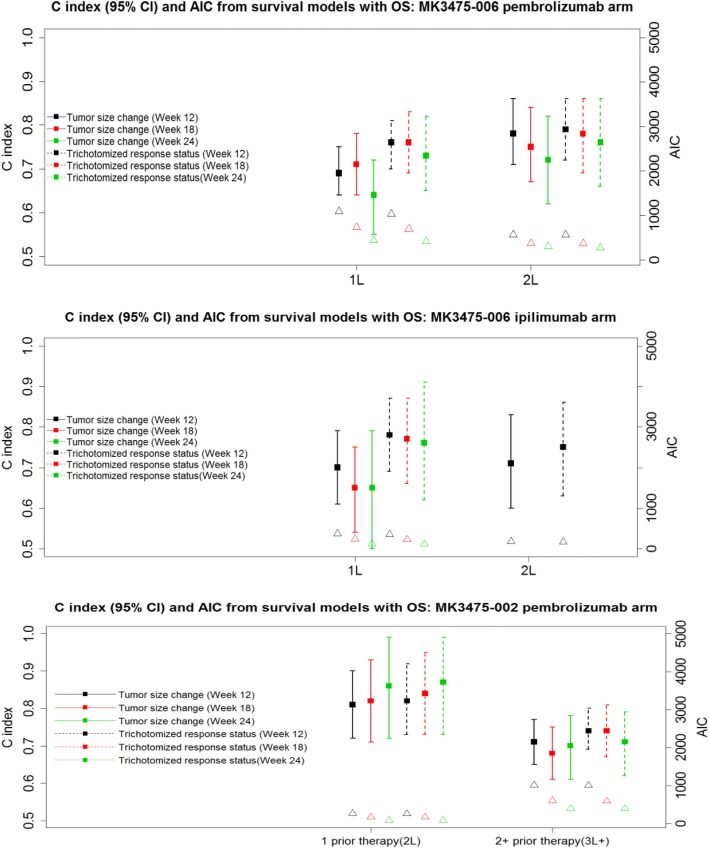
c-index (95%CI) and AIC from Survival Models with OS Based on KEYNOTE-002 and -006. Lines correspond to c-index with 95% CI (leftside of Y axis); Triangles correspond to AIC (rightside of Y axis)

The comparisons of different metrics in terms of HR, c-index and AIC are shown in Fig. 1 and Additional file 3: Table S3 and Additional file 4: Table S4 in the supplement.

After adjusting for baseline tumor sizes, the continuous tumor size change metric and RECIST-based trichotomized response metric at different landmark time points were found to be statistically significantly associated with OS (lower bound of HR 95% CI greater than 1) (third and seventh columns in Additional file 4: Table S4 and Table S4). For example, for 1L patients receiving pembrolizumab from KEYNOTE-006, after adjusting for baseline tumor size, the HR (95%CI) of death for 10% tumor size increase at Week 12 was 1.16 (1.11, 1.20); the HR of death for patients who had PD over PR/CR at Week 12 was 8.41 (5.18, 13.64). The HRs at different landmark time points were greater than 1 with similar values for both tumor size change metric and RECIST-based metric. It is worth noting that analyses adjusting for additional prognostic factors (e.g., ECOG, LDH) were also explored and the results were very similar.

For 1L patients receiving pembrolizumab from KEYNOTE-006, the percentage of tumor size changes (across the different landmarks) appeared to have lower predictive values (c-index) than the RECIST-based trichotomized metric, while it showed similar and comparable predictive values for 2L patients receiving pembrolizumab (top panel in Fig. 1; fifth and ninth columns in Additional file 3: Table S3 for pembrolizumab arm). For example, for 1L the c-index at Week 12 and 18 was 0.69 and 0.71 for tumor size change metric, respectively, and 0.76 and 0.76 for RECIST-based metric, respectively. For 2L the c-index at Week 12 and 18 was 0.78 and 0.75 for tumor size change metric, respectively, and 0.79 and 0.78 for RECIST-based metric, respectively.

For 1L patients receiving ipilimumab from KEYNOTE-006, the percentage of tumor size changes also appeared to have lower predictive values than the RECIST-based trichotomized metric across different time points (middle panel of Fig. 1; fifth column in Additional file 3: Table S3 for ipilimumab arm). For 2L patients, the analysis mainly focuses on early time point Week 12 due to relatively small sample size at later time points. It showed relatively comparable predictive values for 2L patients for both metrics at Week 12 (middle panel of Fig. 1; ninth column in Additional file 3: Table S3 for ipilimumab arm).

For previously heavily treated KEYNOTE-002 patients randomized to pembrolizumab arm, the percentage of tumor size changes showed similar or slightly lower predictive values than the RECIST-based trichotomized metric (bottom panel of Fig. 1; fifth and ninth columns in Additional file 4: Table S4).

It appears the predictive values were not lower at the Week 12 and 18 landmarks than those at the Week 24 time point for both tumor size change metric and RECIST-based trichotomized metric barring cases with small sample size (e.g., Week 24 in patients with 1 prior therapy in KEYNOTE-002) (Fig. 1; fifth and ninth columns in Additional file 3: Table S3 and Additional file 4: Table S4). For example, for 2L patients receiving pembrolizumab from KEYNOTE-006, the c-index for tumor size changes at Week 12, 18 and 24 was 0.78, 0.75 and 0.72, respectively; the c-index for RECIST-based trichotomized metric at Week 12, 18 and 24 was 0.79, 0.78 and 0.76, respectively.

The AIC values from continuous tumor size change metric tended to be smaller at all different time points than those from categorical RECIST-based trichotomized metric (Fig. 1; fourth and eighth columns in Additional file 3: Table S3 and Additional file 4: Table S4).

### Cut-off for early tumor size changes

Recursive partitioning analyses based on either the observed data or bootstrapping suggest that a − 8% change from baseline in tumor size changes at Week 12 may predict survival outcomes. The cutoffs were similar between pooling just pembrolizumab subjects and pooling pembrolizumab and ipilimumab. Additional file 5: Figure S1 in the Supplement shows the survival curves of training datasets based on the cut-off of − 8% tumor size changes at Week 12. The c-indices based on the cut-off values for tumor size changes at other time points (Week 18 and 24) were smaller than 0.73 at Week 12, suggesting Week 12 landmark may be more predictive.

The survival curves of validation datasets (KEYNOTE-001 Melanoma patients) based on the cut-off of − 8% tumor size changes as Week 12 are in Additional file 6: Figure S2 in the Supplement. There was statistically significant difference (*p* < 0.0001) in terms of OS from Week 12 between patients with ETSC ≤ − 8% and those > − 8%. The HR (95% CI) of death for patients with ETSC ≤ − 8% over > − 8% at Week 12 was 0.290 (0.218, 0.385). The 12 month OS (95% CI) estimate from Week 12 for patients with tumor size changes ≤ − 8% and > − 8% was 87% (83, 91%) and 55% (48, 62%), respectively.

Sensitivity analysis based on a range of cut-offs (− 30%, − 20%, − 10%, − 8%, 0%, + 10% and + 20%) using validation datasets shows that c-index is similar for cutoffs with 8 to 30% reduction range (− 8% to − 30% tumor size changes), but relatively lower for other cut-offs (Additional file 7: Figure S3 in the Supplement). Therefore, we rounded up to 10% tumor reduction as an optimal cut-off. It is worth noting c-index is similar to all cut-offs if baseline tumor sizes are adjusted. Additionally, c-index also tends to be higher.

**Fig. 2:**
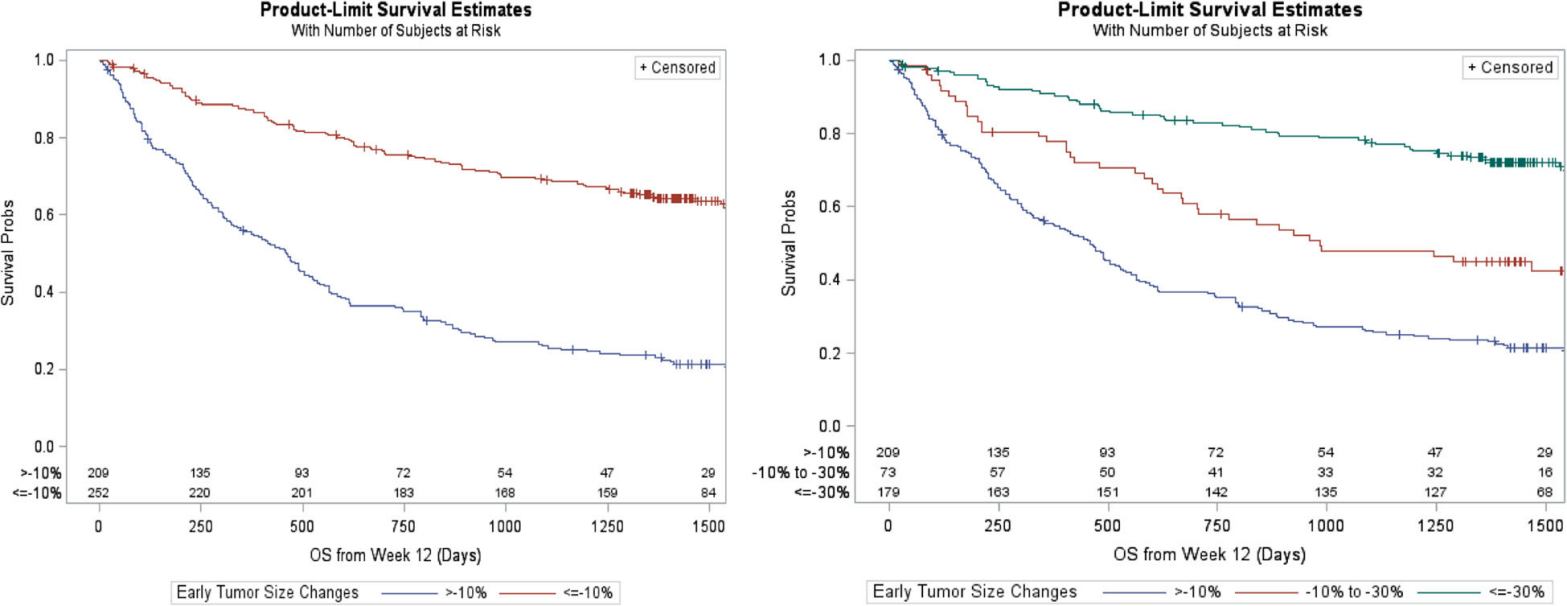
Overall Survival Curves Based on Validation Datasets (KEYNOTE-001 Melanoma Patients). Left panel shows the survival curves with 10% cut-off and the right panel shows survival curves with two cutoffs of 10 and 30% tumor reduction

A “≥10% reduction (tumor size changes ≤-10%)” can be broken down into “10% to 30% reduction” and “≥30% reduction” which corresponds to partial response for target lesions per RECIST. Among 252 patients with ≥10% tumor reduction from validation datasets, 73 and 179 patients had 10% to 30% and ≥ 30% tumor reduction, respectively. Left panel of Fig. 2 shows the survival curves with 10% cut-off and the right panel shows survival curves with two cutoffs of 10% and 30% tumor reduction. There was statistically significant difference (*p* < 0.0001) in terms of OS from Week 12 between patients with tumor size changes > − 10% and ≤ − 10% groups, as well as among > − 10%, − 10% to − 30% and ≤ − 30% groups. It is worth noting the following from validation datasets: approximately 50% of those patients with 10% to 30% tumor reduction had a RECIST PR; 85% of those patents with at least 30% reduction had a RECIST PR; and only 4% of those without a > 10% tumor reduction had a RECIST PR later.

Table 3 shows sensitivity and PPV for surviving additional 1, 2 and 3 years from Week 12 under 3 different cut-offs (− 10%, − 30%, + 20%). The PPV decreases over time, while the sensitivity increases over time under all three different cut-offs. Tumor size changes of − 10% as cut-off appears to have a much higher sensitivity (e.g., 71.2% at 2 years) than tumor size changes of − 30% as cut-off (e.g., 55.3% at 2 years), maintaining a reasonable prediction of survival at all time points (fifth column in Table 3). Tumor size changes of + 20% as cut-off which corresponds to DCR cut-off for target lesions per RECIST, appears to have a very high sensitivity value at all time points (second column in Table 3), but the PPV (third column in Table 3) is low compared to 2 other cut-offs. Specificity and NPVs are listed in Additional file 8: Table S5 in the Supplement.

**Table 3:** Sensitivity and positive predictive value with different tumor size change cut-offs

Survive Additional t (years) from Week 12	+ 20%		−10%		−30%	
	Sensitivity	PPV	Sensitivity^a^	PPV^b^	Sensitivity	PPV
1	92.7%	82.6%	65.4%	87.1%	48.6%	91.0%
2	93.8%	66.2%	71.2%	75.6%	55.3%	82.8%
3	94.9%	57.6%	76.0%	69.1%	60.8%	77.7%

^a^Sensitivity: Prob (tumor size change “<= −10%” at Week 12 given surviving additional t years)

^b^Positive Predictive Value (PPV): Prob(survive additional t years given tumor size change “<= − 10%” at Week 12)

Furthermore, we explored the optimal cut-off via non-parametric kernel density classification method [37]. The results also suggest an approximate − 10% change from baseline at Week 12 may predict survival outcomes.

## Discussions

Due to the unique response pattern of immunotherapeutic agents, there is a need to further explore intermediate endpoints that would better serve as a surrogate endpoint for OS in assessing the clinical benefit and accelerate the drug approval process. We explored if ETSC and early response status were predictive of long-term survival benefit based on 3 melanoma studies with over 1800 patients receiving immunotherapeutic agents.

Our analyses showed tumor size changes were predictive of survival and early tumor size changes (e.g., Week 12) appeared to have reasonable predictive value than other later time points for immunotherapy trials. However, in agreement with earlier research in traditional chemotherapy and target therapy trials, these metrics do not provide any predictive improvement over traditional RECIST response evaluation criteria for immunotherapy trials. The application of the tumor size change cut-off derived from training datasets to validation datasets is successful. This provides evidence not only for the feasibility of the application to other future trials but also the importance of the choice of early time points. Approximately 10% tumor shrinkage for target lesions at an earlier time point (e.g., Week 12) may be a reasonable efficacy screening cut-off while RECIST-based efficacy metrics remains the primary tool, especially for trials with patients in later line of therapies (e.g., 2L+) and when an early decision is desirable. In practice, later time point landmark analysis has limited utility for early efficacy signal detection due to longer follow up time requirement, and relatively smaller and selected patient population at later time points. Under the situation when patient enrollment is fast, early landmark analysis is especially useful for early decision-making and may help program planning and speed up development to get to market earlier. The c-index is a measure of accuracy and serves to ascertain the predictive ability of a given model/metric. However, it is important to note the discriminatory ability of one or more factors for a given outcome depends strictly on the clinical/biology context being considered, as well as on the structure of the model being tested.

Alternative cut-offs have been explored in cancers treated with chemo and target therapies [16, 18–22]. Krajewski et al. [18, 19] identified and validated a 10% tumor shrinkage as a reliable early predictor of OS with a total of 133 mRCC patients receiving VEGF-targeted therapies. Sakamaki et al. [20] suggested a time point of 8 weeks and a cut-off value of 20% are optimal criteria for defining early tumor changes based on 67 patients with metastatic colorectal cancer treated with first-line chemotherapy. Luo et al. and Lamarca et al. [21, 22] identified a 10% tumor reduction to predict progression-free survival based on 33 patients with NETs treated with SSA, and 237 patients with advanced pancreatic neuroendocrine tumors treated with sunitinib, respectively. Based on over 1800 melanoma patients receiving immunotherapeutic agents from 3 studies, we separated data into training and validation datasets. Our analysis suggest 10% tumor reduction at Week 12 may be a reasonable cutoff to be predictive of OS and patients with more than 10% reduction at Week 12 tend to have a longer survival, as well as higher chance of achieving RECIST PR/CR. Compared to the traditional 30% reduction cut-off, 10% reduction as cut-off appears to have higher sensitivity. While the PPV of OS seems higher using traditional 30% reduction cut-off, patients with tumor reduction between 10 and 30% still have good chance of achieving PR/CR and longer survival time. Therefore, it may not be necessary to use a stringent cutoff to serve as early efficacy signal detection. Compared to 20% increase as cut-off, 10% reduction as cut-off appears to have a high specificity and high PPV of OS. Ten percent tumor reduction, which is between 30% tumor reduction for PR/CR and 20% tumor increase for PD, may be the sweet spot to be predictive of OS, also maintaining reasonable sensitivity and specificity.

While 10% reduction at week 12 may be a reasonable efficacy screen cut-off from the perspective of early decision-making and program planning in drug development, patients should be encouraged to stay on treatment until disease progression in case of less than 10% reduction after 12 weeks. Because of the possibility of immunotherapy-related flare, patients who show initial radiographic progression, if they are clinically stable, may continue on treatment at the discretion of the investigator.

ETSC and RECIST-based trichotomized response status should be considered in go/no-go decision process for immunotherapy trials. Based on 3 studies we analyzed, 72% of those patents with more than 10% reduction at Week 12 achieved a RECIST PR/CR, while only 6% of those without a 10% tumor reduction at Week 12 had a RECIST PR later. Approximately 10% tumor reduction may help identify more potential responders, critical for early decision-making process, program planning and design of late-stage trials. Advancing promising therapies earlier is crucial especially within the highly competitive immune-oncology space. ORR and DCR are simple, but the predictive value may decrease with the number of prior therapies. By considering certain amount of tumor reduction (e.g., 10 to 30%) also leads to PR/CR later on and is related with longer survival, composite response rate which takes SD as partially positive outcome and utilize different weights (or utility) for PR/CR and SD, may be of value for go/no-go early decision as well. For example, while a PR/CR is worth a full weight of 1, SD may be worth a partial weight of 0.5 given that approximately half of the patients had a PR later based on 3 studies we analyzed. The optimal weighting strategy that best predicts OS is a research topic beyond of the scope of this paper.

Our analysis has strengths such as the fact that all these 3 studies have long-term survival data, in addition to the quality-assurance of these data for drug registration purpose. Our recommended 10% tumor reduction cutoff is based on separate training and validation datasets, and also validated by different statistical classification approaches. One limitation of our study was inclusion of only one tumor type. Whether our findings in melanoma patients can be generalized to other cancer types should also be explored. Second, our findings are associated with checkpoint blockade based immunotherapy (e.g., pembrolizumab, ipilimumab). Other types of immunotherapy (e.g., oncolytic viruses) may have different treatment response pattern. Therefore, our findings specifically apply to checkpoint blockade based immunotherapy in metastatic melanoma and whether they can be generalized to other immunotherapies is unknown. Another limitation of this research (same as other research using continuous tumor size as the early efficacy metric) is that the early efficacy metrics would only include the numerical values from the size of the target lesions but it would not include data such as response of the non-target lesions or the appearance of new lesions. Further research into other methods that potentially address the whole tumor burden including target, non-target lesion and new lesions should be investigated to better account for therapeutic effects on long-term survival that may be observed in immunotherapy trials.

## Additional files

Additional file 1: **Table S1.** Response Metrics (per RECIST) in Melanoma Patients (Ipilimumab-Naive Melanoma (KEYNOTE -006)). (DOCX 35 kb)

Additional file 2: **Table S2.** Response Metrics (per RECIST) in Melanoma Patients (Ipilimumab-Refractory Melanoma (KEYNOTE -002)). (DOCX 20 kb)

Additional file 3: **Table S3.** Effect and Predictive Value of “Tumor Size Changes” and “RECIST-based Response Status” on OS in Melanoma Patients (Ipilimumab-Naive Melanoma (KEYNOTE -006)). (DOCX 37 kb)

Additional file 4: **Table S4.** Effect and Predictive Value of “Tumor Size Changes” and “RECIST-based Response Status” on OS in Melanoma Patients (Ipilimumab-Refractory Melanoma (KEYNOTE -002)). (DOCX 35 kb)

Additional file 5: **Figure S1.** Early Tumor Size Changes Cut-off Based on KEYNOTE-002 and -006. (DOCX 154 kb)

Additional file 6: **Figure S2.** KEYNOTE-001 Melanoma Patients Overall Survival Curves Based on the Early Tumor Size Changes Cut-off of “-8%” (DOCX 36 kb)

Additional file 7: **Figure S3.** Sensitivity Analysis Under Different Cut-offs* Based on KEYNOTE-001**. (DOCX 50 kb)**

Additional file 8: **Table S5.** Specificity and Negative Predictive Value with Different Tumor Size Change Cut-offs. (DOCX 33 kb)

## Abbreviations

AIC: Akaike information criteria
CR: Complete Response
DCR: Disease Control Rate
ETSC: Early Tumor Size Changes
HR: Hazard Ratio
NPV: Negative Predictive Value
ORR: Objective Response Rate
OS: Overall Survival
PD: Progressive Disease
PFS: Progression-Free Survival
PH: Proportional Hazard
PPV: Positive Predictive Value
PR: Partial Response
RECIST: Response Evaluation Criteria in Solid Tumors
SD: Stable Disease

## References

[1] Eisenhauer E, Therasse P, Bogaerts J, Schwartz LH, Sargent D, Ford R., *et al.*. New response evaluation criteria in solid tumors: revised RECIST guideline (version 1.1). Eur J Cancer, 2009;45(2):228–247. 10.1016/j.ejca.2008.10.026 19097774

[2] Karrison TG, Maitland ML, Stadler WM, Ratain MJ (2007). Design of Phase II Cancer trials using a continuous endpoint of change in tumor size: application to a study of Sorafenib and Erlotinib in non–small-cell NSCLC Cancer. J Natl Cancer Inst.

[3] Jaki T, Andre V, Su TL, Whitehead J (2013). Designing exploratory cancer trials using change in tumour size as primary endpoint. Stat Med.

[4] Suzuki C, Blomqvist L, Sundin A, Jacobsson H, Byström P, Berglund Å (2012). The initial change in tumor size predicts response and survival in patients with metastatic colorectal cancer treated with combination chemotherapy. Ann Oncol.

[5] Piessevaux H, Buyse M, Schlichting M, Van Cutsem E, Bokemeyer C, Heeger S (2013). Use of early tumor shrinkage to predict long-term outcome in metastatic colorectal cancer treated with cetuximab. J Clin Oncol.

[6] An MW, Dong X, Meyers J, Han Y, Grothey A, Bogaerts J (2015). Evaluating Continuous Tumor Measurement-Based Metrics as Phase II Endpoints for Predicting Overall Survival. J Natl Cancer Inst.

[7] An MW, Han Y, Meyers JP, Bogaerts J, Sargent DJ, Mandrekar SJ (2015). Clinical utility of metrics based on tumor measurements in phase II trials to predict overall survival outcomes in phase III trials by using resampling methods. J Clin Oncol.

[8] An MW, Mandrekar SJ, Branda ME, Hillman SL, Adjei AA, Pitot HC (2011). Comparison of continuous vs categorical tumor measurement-based metrics to predict overall survival in cancer treatment trials. Clin Cancer Res.

[9] Claret L, Gupta M, Han K, Joshi A, Sarapa N, He J (2013). Evaluation of tumour-size response metrics to predict overall survival in Western and Chinese patients with first-line metastatic colorectal Cancer. J Clin Oncol.

[10] Taieb J, Rivera F, Siena S, Karthaus M, Valladares-Ayerbes M, Gallego J (2018). Exploratory analyses assessing the impact of early tumour shrinkage and depth of response on survival outcomes in patients with RAS wild-type metastatic colorectal cancer receiving treatment in three randomised panitumumab trials. J Cancer Res Clin Oncol.

[11] Heinemann V, Stintzing S, Modest DP, Giessen-Jung C, Michl M, Mansmann UR (2015). Early tumour shrinkage (ETS) and depth of response (DpR) in the treatment of patients with metastatic colorectal cancer (mCRC). Eur J Cancer.

[12] Lee CK, Kim SS, Park S, Kim C, Heo SJ, Lim JS (2017). Depth of response is a significant predictor for long-term outcome in advanced gastric cancer patients treated with trastuzumab. Oncotarget.

[13] Kaufman H, Schwartz LH, William WN, Sznol M, Aguila M, Whittington C, et al. Evaluation of clinical endpoints as surrogates for overall survival in patients treated with immunotherapies. J Clin Oncol. 35(15_suppl - published online before print). 10.1200/JCO.2017.35.15_suppl.e14557.

[14] Wolchok JD, Hoos A, O'Day S, Weber JS, Hamid O, Lebbé C (2009). Guidelines for the evaluation of immune therapy activity in solid tumors: immune-related response criteria. Clin Cancer Res.

[15] Hodi FS, Hwu WJ, Kefford R, Weber JS, Daud A, Hamid O (2016). Evaluation of immune-related response criteria and RECIST v1.1 in patients with advanced melanoma treated with pembrolizumab. J Clin Oncol.

[16] Blumenthal GM, Zhang L, Zhang H, Kazandjian D, Khozin S, Tang S (2017). Milestone Analyses of Immune Checkpoint Inhibitors, Targeted Therapy, and Conventional Therapy in Metastatic Non-Small Cell Lung Cancer Trials: A Meta-analysis. JAMA Oncol.

[17] Mushti S, Mulkey F, Sridhara R (2018). Evaluation of overall response rate and progression-free survival as potential surrogate endpoints for overall survival in immunotherapy trials. Clin Cancer Res.

[18] Krajewski KM, Franchetti Y, Nishino M, Fay AP, Ramaiya N, Van den Abbeele AD (2014). 10% tumor diameter shrinkage on the first follow-up computed tomography predicts clinical outcome in patients with advanced renal cell carcinoma treated with angiogenesis inhibitors: a follow-up validation study. Oncologist.

[19] Krajewski KM, Guo M, Van den Abbeele AD, Yap J, Ramaiya N, Jagannathan J (2011). Comparison of four early post therapy imaging changes (EPTIC; RECIST 1.0, tumor shrinkage, computed tomography tumor density, Choi criteria) in assessing outcome to vascular endothelial growth factor-targeted therapy in patients with advanced renal cell carcinoma. Eur Urol.

[20] Sakamaki K, Kito Y, Yamazaki K, Izawa N, Tsuda T, Morita S (2017). Exploration of time points and cut-off values for early tumour shrinkage to predict survival outcomes of patients with metastatic colorectal cancer treated with first-line chemotherapy using a biexponential model for change in tumour size. ESMO Open.

[21] Luo Y, Chen J. Optimisation of the Size Variation Threshold for CT Evaluation of Response in Advanced Gastroenteropancreatic Nauroendocrine Tumors Treated with Octreotide LAR. ENETS Annual Coference 2017; abstract K11. 10.1007/s00330-018-5512-1 29876704

[22] Lamarca A, Barriuso J, Kulke M, Borbath I, Lenz HJ, Raoul JL (2018). Determination of an optimal response cut-off able to predict progression-free survival in patients with well-differentiated advanced pancreatic neuroendocrine tumours treated with sunitinib: an alternative to the current RECIST-defined response. Br J Cancer.

[23] Ribas A, Puzanov I, Dummer R, Schadendorf D, Hamid O, Robert C (2015). Pembrolizumab versus investigator-choice chemotherapy for ipilimumab-refractory melanoma (KEYNOTE-002): a randomised, controlled, phase 2 trial. Lancet Oncol.

[24] Robert C, Schachter J, Long GV, Arance A, Grob JJ, Mortier L (2015). Pembrolizumab versus ipilimumab in advanced melanoma. N Engl J Med.

[25] Schachter J, Ribas A, Long GV, Arance A, Grob JJ, Mortier L (2017). Pembrolizumab versus ipilimumab for advanced melanoma: final overall survival results of a multicentre, randomised, open-label phase 3 study (KEYNOTE-006). Lancet.

[26] Ribas A, Hamid O, Daud A, Hodi FS, Wolchok JD, Kefford R (2016). Association of pembrolizumab with tumor response and survival among patients with advanced melanoma. JAMA.

[27] Joseph R, Elassaiss-Schaap J, Kefford R, Hwu W, Wolchok J, Joshua A (2018). Baseline tumor size is an independent prognostic factor for overall survival in patients with melanoma treated with Pembrolizumab. Clin Cancer Res.

[28] Anderson J, Cain K, Gelber R (1983). Analysis of survival by tumor response. J Clin Oncol.

[29] Breiman L, Friedman J, Olshen R, Stone C (1984). Classification and regression trees.

[30] Breiman L (1996). Bagging predictors. Mach Learn.

[31] Blanche P, Dartigues J, Jacqmin-Gadda H (2013). Estimating and comparing time dependent areas under receiver operating characteristic curves for censored event times with competing risks. Stat in med.

[32] Li L, Greene T, Hu B. A simple method to estimate the time-dependent receiver operating characteristic curve and the area under the curve with right censored data. Stat methods Med Res. 2016;27(8):2264-2278. 10.1177/0962280216680239 27895266

[33] Claeskens G, Hjort N. Model Selection and Model Averaging. Cambridge; Cambridge University Press; 2008.

[34] Harrell FE, Califf RM, Pryor DB, Lee KL, Rosati RA (1982). Evaluating the yield of medical tests. JAMA.

[35] Harrell FE, Lee KL, Califf RM, Pryor DB, Lee KL, Rosati RA (1984). Regression modeling strategies for improved prognostic prediction. Stat in Med.

[36] Harrell FE, Lee KL, Mark DB (1996). Tutorial in biostatistics: multivariable prognostic models: issues in developing models, evaluating assumptions and adequacy, and measuring and reducing errors. Stat in Med.

[37] Hastie T, Tibshirani R, Friedman JH (2009). The elements of statistical learning: data mining, inference, and prediction.

