# Supplementary material for: Are tumor size changes predictive of survival for checkpoint blockade based immunotherapy in metastatic melanoma?
Source: J Immunother Cancer. 2019 Feb 8;7:39. doi: 10.1186/s40425-019-0513-4 (PMC6368769; doi:10.1186/s40425-019-0513-4)
Supplement: Supplementary file 1 — Table S1. Response Metrics (per RECIST) in Melanoma Patients (Ipilimumab-Naive Melanoma (KEYNOTE -006)). (DOCX 35 kb) [file 40425_2019_513_MOESM1_ESM.docx]

Table S-1 Response Metrics (per RECIST) in Melanoma Patients
(Ipilimumab-Naive Melanoma (KEYNOTE -006) )

|  | 1L | | 2L | |
| --- | --- | --- | --- | --- |
|  | n | % | n | % |
| **Pembrolizumab arm** |  |  |  |  |
| Trichotomized response status (PR/CR vs. SD vs. PD) (~Week 12) | 327 | 100 | 175 | 100 |
| PR/CR | 119 | **36.4** | 42 | **24.0** |
| SD | 86 | **26.3** | 41 | **23.4** |
| PD | 122 | 37.3 | 92 | 52.6 |
| Best trichotomized response status (PR/CR vs. SD vs. PD) | 326 | 100 | 175 | 100 |
| PR/CR | 148 | **45.4** | 59 | **33.7** |
| SD | 61 | **18.7** | 23 | **13.1** |
| PD | 117 | 35.9 | 93 | 53.1 |
| **Ipilimumab arm** |  |  |  |  |
| Trichotomized response status (PR/CR vs. SD vs. PD) (~Week 12) | 150 | 100 | 72 | 100 |
| PR/CR | 18 | 12.0 | 9 | 12.5 |
| SD | 42 | 28.0 | 20 | 27.8 |
| PD | 90 | 60.0 | 43 | 59.7 |
| Best trichotomized response status (PR/CR vs. SD vs. PD) | 148 | 100 | 72 | 100 |
| PR/CR | 24 | 16.2 | 13 | 18.1 |
| SD | 35 | 23.6 | 16 | 22.2 |
| PD | 89 | 60.1 | 43 | 59.7 |
