# Supplementary material for: Are tumor size changes predictive of survival for checkpoint blockade based immunotherapy in metastatic melanoma?
Source: J Immunother Cancer. 2019 Feb 8;7:39. doi: 10.1186/s40425-019-0513-4 (PMC6368769; doi:10.1186/s40425-019-0513-4)
Supplement: Supplementary file 2 — Table S2. Response Metrics (per RECIST) in Melanoma Patients (Ipilimumab-Refractory Melanoma (KEYNOTE -002)). (DOCX 20 kb) [file 40425_2019_513_MOESM2_ESM.docx]

Table S-2 Response Metrics (per RECIST) in Melanoma Patients
(Ipilimumab-Refractory Melanoma (KEYNOTE -002))

|  | Number of Prior Line of Therapy Received | | | |
| --- | --- | --- | --- | --- |
|  | 1 | | 2+ | |
|  | **(2L)** | | **(3L+)** | |
|  | n | % | n | % |
| **MK-3475 Treatment Group** |  |  |  |  |
| Trichotomized response status (PR/CR vs. SD vs. PD) (~Wk 12) | 88 | 100 | 227 | 100 |
| PR/CR | 23 | **26.1** | 41 | **18.1** |
| SD | 26 | **29.5** | 68 | **30.0** |
| PD | 39 | 44.3 | 118 | 52.0 |
| Best trichotomized response status (PR/CR vs. SD vs. PD) | 88 | 100 | 224 | 100 |
| PR/CR | 29 | **33.0** | 62 | **27.7** |
| SD | 17 | **19.3** | 39 | **17.4** |
| PD | 42 | 47.7 | 123 | 54.9 |
