# Supplementary material for: Are tumor size changes predictive of survival for checkpoint blockade based immunotherapy in metastatic melanoma?
Source: J Immunother Cancer. 2019 Feb 8;7:39. doi: 10.1186/s40425-019-0513-4 (PMC6368769; doi:10.1186/s40425-019-0513-4)
Supplement: Supplementary file 3 — Table S3. Effect and Predictive Value of “Tumor Size Changes” and “RECIST-based Response Status” on OS in Melanoma Patients (Ipilimumab-Naive Melanoma (KEYNOTE -006)). (DOCX 37 kb) [file 40425_2019_513_MOESM3_ESM.docx]

Table S-3 Effect and Predictive Value of “Tumor Size Changes” and “RECIST-based Response Status” on OS in Melanoma Patients

(Ipilimumab-Naive Melanoma (KEYNOTE -006))

|  | 1L | | | | | 2L | | | | | |
| --- | --- | --- | --- | --- | --- | --- | --- | --- | --- | --- | --- |
|  | n(event) | | HR (95%CI) | AIC | c-index(95%CI) | n(event) | | HR(95%CI) | AIC | | c-index(95%CI) |
| **Pembrolizumab arm *(n=368 for 1L and n=187 for 2L)*** | | | | | | | | | | | |
| **Tumor size changes at Week 12 (10% increase)** | 258(108) | | 1.16 [1.11, 1.20] | 1096 | 0.69 [0.64, 0.75] | 133(69) | 1.21 [1.16, 1.26] | | 567 | | 0.78 [0.71, 0.86] |
| **Tumor size changes at Week 18 (10% increase)** | 210(77) | | 1.21 [1.15, 1.27] | 736 | 0.71 [0.64, 0.78] | 103(47) | 1.19 [1.14, 1.25] | | 373 | | 0.75 [0.67, 0.84] |
| **Tumor size changes at Week 24 (10% increase)** | 176(46) | | 1.18 [1.10, 1.26] | 441 | 0.64 [0.55, 0.72] | 92(37) | 1.08 [1.04, 1.11] | | 306 | | 0.72 [0.62, 0.82] |
| **Trichotomized response status at Week 12** | 255(107) | |  |  |  | 133(69) |  | |  | |  |
| PD |  | | 8.41 [5.18, 13.64] | 1032 | 0.76 [0.70, 0.81] |  | 14.71 [6.19, 35.00] | | 563 | | 0.79 [0.72, 0.86] |
| SD |  | | 1.52 [0.81, 2.85] | 1032 | 0.76 [0.70, 0.81] |  | 4.32 [1.67, 11.19] | | 563 | | 0.79 [0.72, 0.86] |
| PR/CR |  | |  |  |  |  |  | |  | |  |
| **Trichotomized response status at Week 18** | 208(76) | |  |  |  | 103(47) |  | |  | |  |
| PD |  | | 9.49 [5.66, 15.92] | 694 | 0.76 [0.69, 0.83] |  | 11.62 [4.76, 28.33] | | 371 | | 0.78 [0.69, 0.86] |
| SD |  | | 1.14 [0.49, 2.67] | 694 | 0.76 [0.69, 0.83] |  | 4.59 [1.64, 12.84] | | 371 | | 0.78 [0.69, 0.86] |
| PR/CR |  | |  |  |  |  |  | |  | |  |
| **Trichotomized response status at Week 24** | *175(46)* | |  |  |  | 91(36) |  | |  | |  |
| PD |  | | 7.48 [4.11, 13.61] | 418 | 0.73 [0.65, 0.82] |  | 8.70 [3.82, 19.80] | | 281 | | 0.76 [0.66, 0.86] |
| SD |  | | 0.53 [0.12, 2.29] | 418 | 0.73 [0.65, 0.82] |  | 3.57 [1.29, 9.89] | | 281 | | 0.76 [0.66, 0.86] |
| PR/CR |  | |  |  |  |  |  | |  | |  |
| ***Ipilimumab arm (n=181 for 1L and n=97 for 2L)*** | | | | | | | | | | | |
| **Tumor size changes at Week 12 (10% increase)** | 98(45) | 1.14 [1.06, 1.23] | | 372 | 0.7 [0.61, 0.79] | 51(26) | 1.12 [1.06, 1.18] | | | 177 | 0.71 [0.60, 0.83] |
| **Tumor size changes at Week 18 (10% increase)** | 75(30) | 1.10 [1.02, 1.19] | | 240 | 0.65 [0.54, 0.75] | 30(7) | NA | | | NA | NA |
| **Tumor size changes at Week 24 (10% increase)** | 53(16) | 1.10 [1.00, 1.21] | | 122 | 0.65 [0.50, 0.79] | 22(3) | NA | | | NA | NA |
| **Trichotomized response status at Week 12** | 97(45) |  | |  |  | 51(26) |  | | |  |  |
| PD |  | 22.54 [3.06, 165.97] | | 351 | 0.78 [0.69, 0.87] |  | 17.67 [2.32, 134.54] | | | 171 | 0.75 [0.63, 0.86] |
| SD |  | 5.29 [0.67, 41.83] | | 351 | 0.78 [0.69, 0.87] |  | 4.47 [0.51, 39.23] | | | 171 | 0.75 [0.63, 0.86] |
| PR/CR |  |  | |  |  |  |  | | |  |  |
| **Trichotomized response status at Week 18** | 75(30) |  | |  |  | 30(7) |  | | |  |  |
| PD |  | 12.58 [2.91, 54.33] | | 226 | 0.77 [0.66, 0.87] |  | NA | | | NA | NA |
| SD |  | 3.82 [0.79, 18.40] | | 226 | 0.77 [0.66, 0.87] |  | NA | | | NA | NA |
| PR/CR |  |  | |  |  |  |  | | |  |  |
| **Trichotomized response status at Week 24** | 53(16) |  | |  |  | 22(3) |  | | |  |  |
| PD |  | 4.55 [1.28, 16.20] | | 116 | 0.76 [0.62, 0.91] |  | NA | | | NA | NA |
| SD |  | 0.49 [0.05, 4.74] | | 116 | 0.76 [0.62, 0.91] |  | NA | | | NA | NA |
| PR/CR |  |  | |  |  |  |  | | |  |  |
|  |  |  | |  |  |  |  | | |  |  |
