# Supplementary material for: Are tumor size changes predictive of survival for checkpoint blockade based immunotherapy in metastatic melanoma?
Source: J Immunother Cancer. 2019 Feb 8;7:39. doi: 10.1186/s40425-019-0513-4 (PMC6368769; doi:10.1186/s40425-019-0513-4)
Supplement: Supplementary file 4 — Table S4. Effect and Predictive Value of “Tumor Size Changes” and “RECIST-based Response Status” on OS in Melanoma Patients (Ipilimumab-Refractory Melanoma (KEYNOTE -002)). (DOCX 35 kb) [file 40425_2019_513_MOESM4_ESM.docx]

Table S-4 Effect and Predictive Value of “Tumor Size Changes” and “RECIST-based Response Status” on OS in Melanoma Patients

(Ipilimumab-Refractory Melanoma (KEYNOTE -002))

|  | Received “1” prior therapy (2L) | | | | | Received “2+” prior therapy (3L+) | | | | |
| --- | --- | --- | --- | --- | --- | --- | --- | --- | --- | --- |
|  | n(event) | | HR (95%CI) | AIC | c-index(95%CI) | n(event) | HR(95%CI) | | AIC | c-index(95%CI) |
| **Pembrolizumab arm (n=95 for 2L and n=265 for 3L+)** | | | | | | | | | | |
| **Tumor size changes at Week 12 (10% increase)** | 70(41) | 1.62 [1.39, 1.88] | | 264 | 0.81 [0.72, 0.90] | 171(115) | | 1.18 [1.13, 1.24] | 1016 | 0.71 [0.65, 0.77] |
| **Tumor size changes at Week 18 (10% increase)** | 56(28) | 1.55 [1.33, 1.80] | | 165 | 0.82 [0.71, 0.93] | 121(72) | | 1.09 [1.05, 1.14] | 609 | 0.68 [0.61, 0.75] |
| **Tumor size changes at Week 24 (10% increase)** | 43(17) | 1.68 [1.37, 2.07] | | 89 | 0.86 [0.72, 1.01] | 101(48) | | 1.20 [1.09, 1.31] | 392 | 0.7 [0.61, 0.78] |
| **Trichotomized response status at Week 12** | 70(41) |  | |  |  | 171(115) | |  |  |  |
| PD |  | 45.50 [10.13, 204.35] | | 261 | 0.82 [0.73, 0.92] |  | | 4.58 [2.67, 7.85] | 1006 | 0.74 [0.69, 0.80] |
| SD |  | 7.02 [1.53, 32.17] | | 261 | 0.82 [0.73, 0.92] |  | | 1.20 [0.64, 2.24] | 1006 | 0.74 [0.69, 0.80] |
| PR/CR |  |  | |  |  |  | |  |  |  |
| **Trichotomized response status at Week 18** | 56(28) |  | |  |  | 121(72) | |  |  |  |
| PD |  | 43.43 [9.52, 198.08] | | 166 | 0.84 [0.73, 0.95] |  | | 5.51 [2.95, 10.26] | 590 | 0.74 [0.67, 0.81] |
| SD |  | 11.26 [2.34, 54.24] | | 166 | 0.84 [0.73, 0.95] |  | | 2.00 [0.99, 4.03] | 590 | 0.74 [0.67, 0.81] |
| PR/CR |  |  | |  |  |  | |  |  |  |
| **Trichotomized response status at Week 24** | 43(17) |  | |  |  | 100(48) | |  |  |  |
| PD |  | 72.67 [8.93, 591.51] | | 88 | 0.87 [0.73, 1.00] |  | | 3.69 [1.90, 7.16] | 392 | 0.71 [0.62, 0.79] |
| SD |  | 25.15 [2.76, 229.47] | | 88 | 0.87 [0.73, 1.00] |  | | 1.88 [0.86, 4.10] | 392 | 0.71 [0.62, 0.79] |
| PR/CR |  |  | |  |  |  | |  |  |  |
