# Supplementary material for: Are tumor size changes predictive of survival for checkpoint blockade based immunotherapy in metastatic melanoma?
Source: J Immunother Cancer. 2019 Feb 8;7:39. doi: 10.1186/s40425-019-0513-4 (PMC6368769; doi:10.1186/s40425-019-0513-4)
Supplement: Supplementary file 5 — Figure S1. Early Tumor Size Changes Cut-off Based on KEYNOTE-002 and -006. (DOCX 154 kb) [file 40425_2019_513_MOESM5_ESM.docx]

Figure S-1 Early Tumor Size Changes Cut-off Based on KEYNOTE-002 and -006


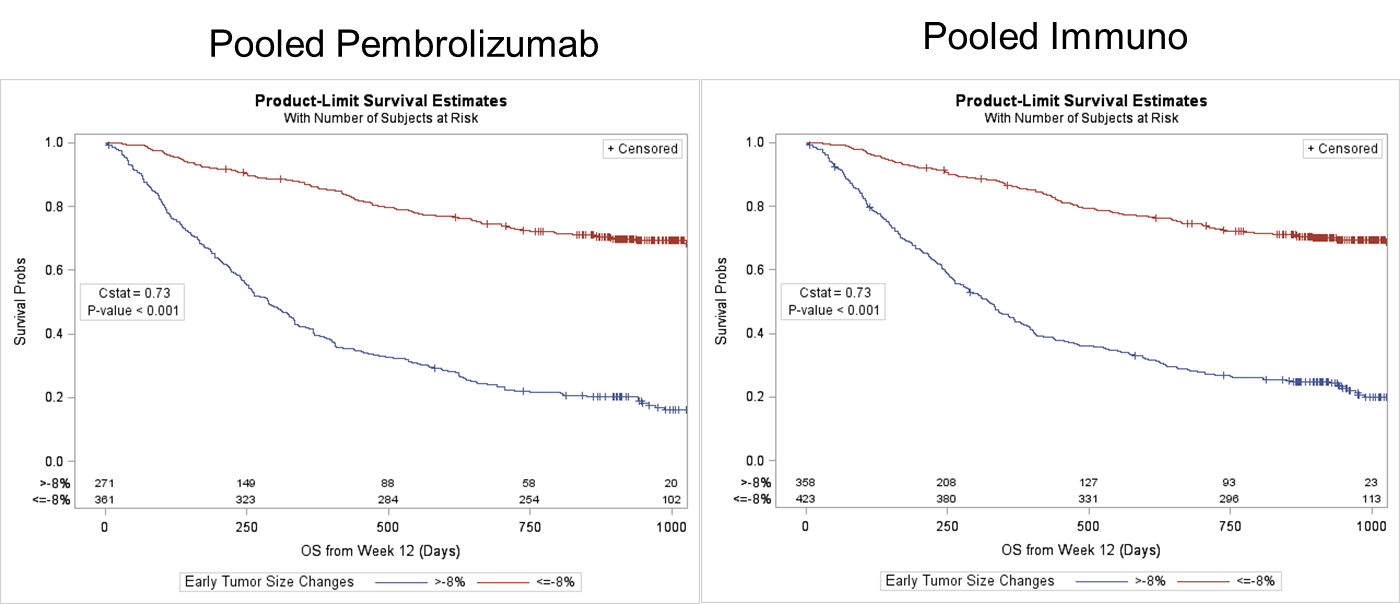


“Pooled Immuno” means pooling pembrolizumab and ipilimumab.
