# Supplementary material for: Are tumor size changes predictive of survival for checkpoint blockade based immunotherapy in metastatic melanoma?
Source: J Immunother Cancer. 2019 Feb 8;7:39. doi: 10.1186/s40425-019-0513-4 (PMC6368769; doi:10.1186/s40425-019-0513-4)

Figure S-2 KEYNOTE-001 Melanoma Patients Overall Survival Curves Based on the Early Tumor Size Changes Cut-off of “-8%”


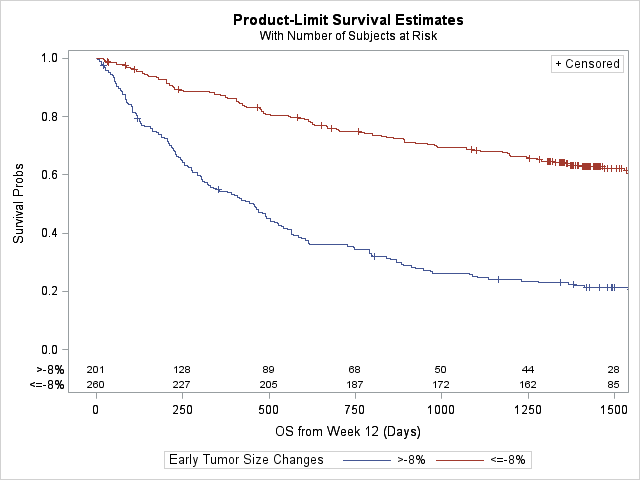

Supplement: Supplementary file 6 — Figure S2. KEYNOTE-001 Melanoma Patients Overall Survival Curves Based on the Early Tumor Size Changes Cut-off of “-8%” (DOCX 36 kb) [file 40425_2019_513_MOESM6_ESM.docx]
