# Supplementary figures and images for: Are tumor size changes predictive of survival for checkpoint blockade based immunotherapy in metastatic melanoma?
Source: J Immunother Cancer. 2019 Feb 8;7:39. doi: 10.1186/s40425-019-0513-4 (PMC6368769; doi:10.1186/s40425-019-0513-4)

Figure S-3 Sensitivity Analysis Under Different Cut-offs* Based on KEYNOTE-00**1**


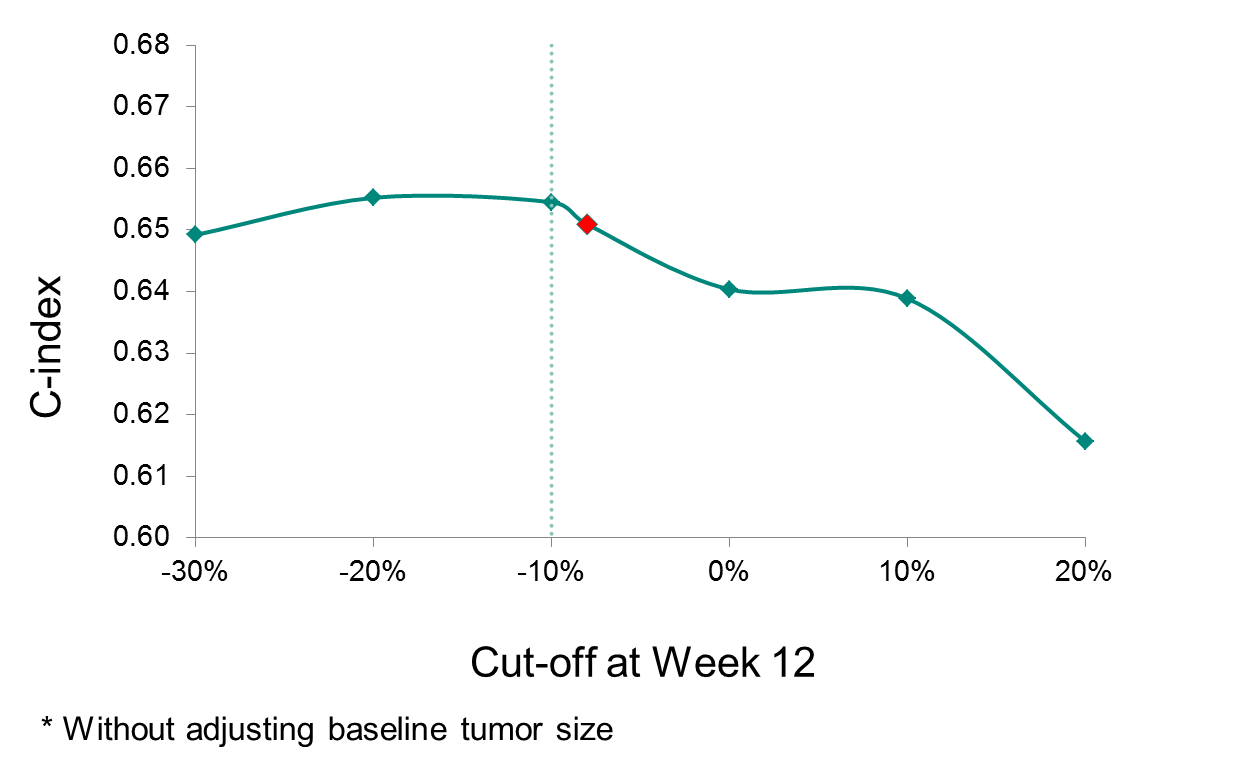

Supplement: Supplementary file 7 — Figure S3. Sensitivity Analysis Under Different Cut-offs* Based on KEYNOTE-001. (DOCX 50 kb) [file 40425_2019_513_MOESM7_ESM.docx]
