# Supplementary material for: Are tumor size changes predictive of survival for checkpoint blockade based immunotherapy in metastatic melanoma?
Source: J Immunother Cancer. 2019 Feb 8;7:39. doi: 10.1186/s40425-019-0513-4 (PMC6368769; doi:10.1186/s40425-019-0513-4)
Supplement: Supplementary file 8 — Table S5. Specificity and Negative Predictive Value with Different Tumor Size Change Cut-offs. (DOCX 33 kb) [file 40425_2019_513_MOESM8_ESM.docx]

Table S-5 Specificity and Negative Predictive Value with Different Tumor Size Change Cut-offs

| Die within next t (years) from Week 12 | +20% | | -10% | | -30% | |
| --- | --- | --- | --- | --- | --- | --- |
|  | Specificity | NPV | Specificity* | NPV** | Specificity | NPV |
| 1 | 47.56% | 70.90% | 74.18% | 44.45% | 87.09% | 38.79% |
| 2 | 34.78% | 80.38% | 68.68% | 63.63% | 84.35% | 58.03% |
| 3 | 31.40% | 86.32% | 66.55% | 73.89% | 82.83% | 68.30% |
| *Specificity: Prob (tumor size change “> -10%” at Week 12 given dying within next t years)  **Negative Predictive Value (NPV): Prob(die within next t years given tumor size change “> -10%” at Week 12) | | | | | | |
